# Creatine Use Among Women Across Reported Duration-of-Use Groups: A Platform-Based Cross-Sectional Survey of Supplementation Behaviors, Motivations, Knowledge, and Experiences

**DOI:** 10.3390/nu18182960

**Published:** 2026-09-09

**Authors:** Jocelyn Burridge, Ziyang Zhang, Ali Boolani, Cory Ambrose, Daniel K. Sodickson, Jordan M. Glenn

**Affiliations:** 1 SuppCo Inc., New York, NY 10016, USA; jordan.mckenzie.glenn@gmail.com; 2 Human Performance and Nutrition Research Institute, Oklahoma State University, Stillwater, OK 74078, USA; 3 Function Health, Austin, TX 78701, USA; 4 Department of Human Physiology, Boston University, Boston, MA 02215, USA

**Keywords:** creatine, women’s health, dietary supplements, cognition, healthy aging, confidence, consumer behavior, supplement use, consumer health information

## Abstract

Background: Creatine is now discussed beyond sports performance and within women’s health, cognition, fatigue, and healthy-aging contexts. However, little is known about the characteristics of women who use creatine or whether these characteristics differ across durations of use. This study characterized women reporting current or past-12-month creatine use in a large cross-sectional sample and benchmarked findings against men. Methods: In a cross-sectional, supplement-platform convenience survey (approximately 2.0% response rate; a secondary analysis of a previously reported cohort), 2094 current or recent creatine users (1055 women, 1039 men; mean age 53 years [54.0 in women and 52.5 in men]) reported duration of use, supplementation goals, information sources, creatine knowledge alignment using a five-item battery, self-rated confidence, satisfaction, and intent to continue supplementation for another six months. Representation of women across six ordered duration bands was evaluated using an age-adjusted logistic model with duration as a categorical factor, with a Cochran–Armitage trend test reported alongside it. Among women, goals and information sources were compared across duration-of-use bands using unadjusted tests of homogeneity, and knowledge alignment was evaluated using an ordered trend test. Gender differences were estimated in age- and duration-adjusted models, pooled across duration groups, with Benjamini–Hochberg correction within domain; gender × duration interactions were tested for the goal and information-source outcomes. Self-rated confidence was modeled as a function of knowledge, gender, and their interaction. Results: Women comprised 50.0–63.9% of respondents across each reported duration category below three years but only 19.7% of those reporting 3+ years of use. In the primary age-adjusted analysis, gender composition differed strongly across duration categories (global *p* < 0.001); relative to the 3+ year group, the odds of identifying as a woman were 3.9- to 7.5-fold higher in every shorter-duration category. Among women, knowledge alignment was higher at longer reported durations in the unadjusted analysis and remained associated with duration after adjustment for age, BMI, and physical activity (global categorical test, *p* < 0.001). Lower knowledge alignment among shorter-duration women reflected greater uncertainty rather than greater myth endorsement, which remained rare across duration groups. Compared with men, women were less likely to report athletic performance as a goal (adjusted 37.5% vs. 50.2%) and more likely to report healthy aging, while six of seven information sources differed by gender. Women also had lower knowledge-alignment scores overall, yet the largest gender difference emerged in confidence: at equivalent observed knowledge alignment, women reported progressively lower self-rated confidence than men as scores increased (interaction *p* = 0.012), with an adjusted difference of −0.38 points at the highest observed knowledge score. Conclusions: Among current and recent SuppCo users who responded to this survey, women showed a pattern of creatine use that differed from the historically performance-focused populations underlying much of the evidence base. Women were disproportionately represented in shorter reported duration-of-use categories, more often endorsed healthy aging and less often athletic performance, and reported different information sources than men. Lower knowledge alignment among shorter-duration women reflected greater uncertainty rather than greater endorsement of misconceptions, while women reported lower confidence than men at the highest observed knowledge levels. These findings suggest future research and education should go beyond efficacy trials in women to address who is using creatine, the outcomes they expect, where they obtain information, and how knowledge and confidence shape decision-making. Prospective studies are needed to determine what underlies the difference in women’s representation across reported duration-of-use groups.

## 1. Introduction

Creatine monohydrate is one of the most extensively studied dietary supplements in sports nutrition [1,2]. It helps muscles store more phosphocreatine, allowing them to quickly regenerate ATP during high-intensity activity. Benefits are well established for high-intensity performance, lean mass, and recovery in athletic populations of men [1,2]. Sex-specific physiology relevant to women’s health has emerged as a new area of interest for creatine action. Some reports suggest approximately 20% lower endogenous creatine synthesis and 30% to 40% lower dietary creatine intake in females than in males [3].

Hormonal variation across menstruation, pregnancy, and menopause may further alter creatine kinetics and substrate availability, with potential downstream effects on muscle, bone, mood, and cognition [3,4]. Three phases of female reproductive life place shifting demands on the body’s creatine system: the monthly rise and fall of estrogen and progesterone across the menstrual cycle, the higher creatine demands of pregnancy, and the drop in estrogen after menopause [3,4,5]. Each has been linked to changes in how much creatine the body makes, how well cells take it up, and how much is stored in muscle. Estrogen appears to drive some of these effects, since it helps regulate two key proteins: the creatine transporter, which moves creatine into cells, and creatine kinase, the enzyme that puts it to work [3,4,6]. The result may point to less creatine available at the life stages when muscle, bone, mood, and cognition may benefit most [3,4]. These observations have motivated hypotheses that creatine supplementation may have life-stage-specific relevance for women [3,4,5].

Despite growing clinical interest, trial evidence in women remains limited and heterogeneous. A 2025 systematic review of 27 studies in active women found improvements relative to placebo in only 3 of 11 strength or power trials and 4 of 17 anaerobic-performance trials; some studies contributed to both outcome categories, and most were limited by small samples and short follow-up [7]. In the largest randomized trial in postmenopausal females to date (N = 237), two years of creatine supplementation did not improve femoral-neck bone mineral density, the primary outcome, although some secondary measures of bone geometry favored creatine supplementation [8]. Meta-analyses suggest small and inconsistent effects on lean mass, with more consistent strength benefits in older women when creatine is combined with resistance training for at least 24 weeks [9,10]. Smaller studies in peri- and postmenopausal females have reported improvements in selected strength, cognitive, and symptom outcomes, but definitive trials remain lacking [11].

Although these studies help define where creatine has been tested in women, they provide little insight into the women who are already using creatine outside research settings. As interest in creatine expands beyond sports performance into healthy aging, cognition, and women’s health, the user population may encompass women with different goals, information sources, levels of knowledge and confidence, and reported durations of use. Understanding this heterogeneity is important because the priorities and expectations of women using creatine may not align with the populations and outcomes that have historically dominated the evidence base. The present study therefore examines how these characteristics vary across reported duration-of-use groups among people identifying as women and how those patterns compare with men.

This study is a secondary analysis of the same SuppCo survey and cohort reported previously in Reference [12] and draws on the same respondents. The companion report characterized the cohort overall, including supplementation behaviors, goals and perceived benefits, knowledge and misconceptions, side effects, satisfaction, and demographic comparisons, but did not examine heterogeneity among women across reported duration of creatine use. This is an important distinction because overall cohort summaries can obscure whether women at different reported durations represent different user profiles, with different goals, information pathways, knowledge, and confidence. The present study therefore uses reported duration as the organizing framework to examine women’s representation across duration-of-use groups and to characterize how these profiles vary across duration and relative to men within the same cohort. This duration-structured approach is intended to identify differences that may be relevant to women-focused research, education, and expectation-setting but are not visible in aggregate descriptions of creatine users.

## 2. Materials and Methods

### 2.1. Study Design and Participants

This secondary cross-sectional analysis of the same underlying survey dataset reported in Reference [12] used a convenience sample recruited through the SuppCo platform (New York, NY, USA) between January and April 2026 to characterize gender differences across creatine duration-of-use groups, with particular emphasis on the characteristics of women who use creatine. Recruitment, consent procedures, and the survey instrument have been described in prior work [12].

The survey instrument was developed and internally reviewed by the research team and pilot tested with 10 individuals without formal scientific training to refine wording and clarity before distribution. Of approximately 3300 users who began the survey, 2233 submitted questionnaires (approximately 68%); median completion time was 16 min and 3 s. All substantive items were optional, unsubmitted survey sessions were not retained, and responses could not be revised after submission. Respondents were instructed to submit one response; because no account identifier was retained in the analytic dataset, duplicate submissions could not be definitively identified.

Eligibility required current or past-12-month creatine use. Gender-group analyses were restricted to respondents identifying as women or men. Respondents selecting “I cycle on and off, unsure of total duration” were treated as providing a valid but nonordinal duration response and were excluded only from analyses requiring placement on the six-level ordered duration axis. Reported daily doses > 30 g/day were treated as implausible and set to missing for dose-specific analyses only; respondents were retained in analyses not involving dose. Participant flow is shown in Supplementary Figure S1.

Survey responses were collected without linkage to participants’ SuppCo account records. Date of birth, height, and weight were collected within the research survey to derive age and BMI; date of birth was not retained in the analytic dataset used for statistical analysis. The optional email field used to request a summary of study findings was collected separately, was not linked to individual survey responses, and was not included in the analytic dataset.

Reference [12] analyzed the same underlying survey cohort and reported overall supplementation and dosing patterns, goals and perceived benefits, creatine knowledge and misconceptions, side effects, satisfaction, continuation intention, information needs, and selected comparisons by gender and age. The present analytic samples reflect the duration-related and model-specific restrictions described above and in Supplementary Table S1. Analyses unique to the present manuscript include age-adjusted categorical modeling of women’s representation across all six reported duration categories, duration-structured within-women analyses with covariate-adjusted sensitivity analyses, gender × duration interaction analyses, and evaluation of the gender × knowledge-alignment interaction in self-rated confidence.

### 2.2. Measures

#### 2.2.1. Participant Characteristics

Demographics. Age was calculated from date of birth, with categorical age strata used for stratified robustness analyses (18–29, 30–49, 50–64, 65–79, 80+ years). Gender identity was self-reported (woman, man, nonbinary, prefer not to say) and dichotomized as women versus men. Participants therefore self-reported gender identity rather than biological sex, and the terms “women” and “men” are used throughout. Sex assigned at birth, menstrual status, pregnancy or lactation status, and menopausal status were not measured, and no inference about biological sex is implied. Body mass index (BMI) was derived from self-reported height and weight; implausible values (<15 or >60 kg/m^2^) were set to missing.

Duration of creatine use. Respondents self-reported their total duration of creatine use (“How long have you used creatine (in total)?”) by selecting among seven categories, including six ordinal levels (<1 month, 1–3 months, 3–6 months, 6–12 months, 1–3 years, 3+ years) and a non-ordinal cycling option (“I cycle on and off, unsure of total duration”). The six ordered levels were coded 1 through 6. Four grouped duration categories (≤3 months, 3–12 months, 1–3 years, and 3+ years) were defined a priori to support pooled gender comparisons.

#### 2.2.2. User Characteristics

Supplementation behaviors. Respondents reported typical daily dose (g), dosing frequency (once daily, multiple times daily, a few times per week, only during training phases, or inconsistently), and prior use of a loading phase (yes, no, or “not sure what a loading phase is”). Daily doses exceeding 30 g/day were treated as implausible (*n* = 7; all excluded doses were above 250 g/day).

Goals for creatine use. Respondents selected from a checklist of 11 goals (muscle strength, muscle mass, athletic performance, exercise recovery, injury recovery, cognitive performance, energy or fatigue reduction, healthy aging, weight management, general wellness, medical or clinical reasons). Each goal was treated as a binary indicator. The two goals endorsed by fewer than 5% of respondents (injury recovery and medical or clinical reasons) were excluded, leaving nine modeled goals.

Information sources. Respondents reported how they first learned about creatine using a check-all-that-apply checklist of eight options (coach or trainer, friend or family, social media, YouTube, podcasts, healthcare provider, research article, SuppCo). The SuppCo source was excluded from analyses as a survey-distribution artifact, leaving seven sources.

Creatine knowledge alignment. Five brief statements assessed alignment between user beliefs and current scientific consensus, each keyed as “false”: “creatine causes kidney damage,” “creatine causes hair loss,” “creatine is a steroid,” “creatine is only beneficial for people who lift weights,” and “long-term creatine use is unsafe.” The reference population for these health- and safety-related items was generally healthy individuals using creatine at doses represented in the published human literature. The keyed answers reflect that long-term supplementation has not been associated with adverse renal, hepatic, or other outcomes at recommended doses in controlled trials and position-stand reviews [1,2,13]; that a recent randomized controlled trial found no causal effect on hair loss [13,14]; that creatine is biochemically distinct from anabolic-androgenic steroids [13]; and that benefits extend beyond resistance-training contexts, including cognitive and healthy-aging applications [2,13]. A sixth candidate statement, “creatine causes water retention,” was not scored because its accuracy is dose- and context-dependent and cannot be classified unambiguously; this decision was made during analytic refinement rather than prespecified, and the item was likewise excluded from the companion analysis of the same survey [12]. Responses (“true,” “false,” or “unsure”) were coded 1 for correct and 0 otherwise and summed to a 0–5 knowledge-alignment score. Internal consistency was moderate (KR-20 = 0.67).

Self-rated confidence. Respondents reported confidence in their understanding of how creatine works on a 5-point Likert scale (1 = not at all confident to 5 = extremely confident). 

#### 2.2.3. User Experience

Satisfaction with creatine was rated on a 1–10 scale.

Continuation intention (intent to continue supplementation for six months) was captured as “yes,” “no,” or “unsure” and dichotomized as “yes” versus otherwise. The six-month continuation measure reflects respondents’ stated intention to continue rather than observed subsequent persistence.

### 2.3. Statistical Analysis

**Distribution of women across duration-of-use groups.** Gender composition across the six ordered duration bands was evaluated using age-adjusted logistic regression, with being a woman as the outcome and duration entered as a six-level categorical factor with the 3+ year band as reference assessed by likelihood-ratio test. Duration was modeled categorically because the bands differ substantially in width; a Cochran–Armitage trend test across the ordered bands is reported alongside it. Robustness to the functional form of age was assessed using a natural-spline specification, and representation was additionally tabulated within five age strata. This analysis was designated as the primary analytic question; the hierarchy was not preregistered and should not be interpreted as confirmatory. The analytic sample size was fixed by the number of eligible submitted questionnaires rather than determined by an a priori power calculation. All remaining analyses were treated as secondary and exploratory.

**Characteristics of women across duration of use.** Among women, supplementation goals, information sources, knowledge alignment, and myth endorsement were compared across the six ordered duration bands; supplementation behaviors were summarized descriptively. Because several binary-outcome trajectories were non-monotone, chi-square tests of homogeneity were used, with Monte Carlo *p*-values (10,000 replicates) where expected cell counts fell below five. Ordered differences in the 0–5 knowledge-alignment score were evaluated using the Jonckheere–Terpstra test, with Spearman rank correlation reported as a descriptive measure of ordered association. Benjamini–Hochberg false-discovery-rate correction was applied separately within the goal, information-source, and belief-item domains. These analyses were unadjusted and describe the duration groups as observed.

As a covariate-adjusted sensitivity analysis, goal, information-source, and knowledge-alignment across reported duration were evaluated with adjustment for age, BMI, and physical-activity level. Reported duration was entered as a six-level categorical factor, and the duration effect was assessed with a global likelihood-ratio test for binary outcomes or a global F-test for knowledge alignment. Benjamini–Hochberg correction was applied within domain; results are reported in Supplementary Table S9.

**Benchmarking against men.** Gender differences were estimated using one model per outcome, adjusted for four-level duration group and age. Binary outcomes were analyzed using logistic regression and reported as odds ratios with 95% confidence intervals; the knowledge-alignment score was analyzed using linear regression and reported as a regression coefficient with a 95% confidence interval. Benjamini–Hochberg correction was applied separately within the goal and information-source domains. Gender × duration-group interactions were tested for the nine goal and seven information-source outcomes using likelihood-ratio tests, with Benjamini–Hochberg correction across the 16 interaction tests; these analyses are reported in the Supplementary Table S5. Pooled estimates were used as the primary overall gender comparisons. Self-rated confidence was analyzed using linear regression with knowledge-alignment score centered at 5, gender, their interaction, duration group, and age as predictors. Dose, dosing frequency, loading-phase responses, satisfaction, and continuation intention were summarized descriptively by gender and duration band. 

Models used complete cases for the variables included in each analysis; no imputation was performed. Continuous variables were summarized using the mean and standard deviation when approximately symmetric (age) and the median and interquartile range when skewed (BMI and daily creatine dose). Distributional form was assessed by inspection rather than formal normality testing. For linear models, assumptions were evaluated using model residuals rather than the raw variables. Across inferential analyses, 57 primary and secondary outcome tests were conducted. Sensitivity and model-diagnostic procedures were not counted as independent discovery tests because they assessed robustness of the same hypotheses rather than additional outcomes. Benjamini–Hochberg correction was applied separately within the goal, information-source, and belief-item domains, with the gender × duration interaction tests treated as a separate family. Other sensitivity analyses were considered exploratory.

**Sensitivity analyses.** Robustness of the confidence model was assessed using HC3 heteroscedasticity-robust standard errors, proportional-odds ordinal regression treating confidence as ordered, and a categorical specification of knowledge alignment. The bounded 0–5 knowledge-alignment score was additionally evaluated using proportional-odds ordinal and quasi-binomial models, and Rasch and two-parameter logistic item-response-theory scores were examined as alternative scoring specifications. For the within-women performance-related goal models, an additional sensitivity analysis added strength-training participation to the age-, BMI-, and physical-activity-level-adjusted models.

**Software and Ethics.** Analyses were conducted in R (version 4.4.0; R Foundation for Statistical Computing, Vienna, Austria). All participants provided electronic informed consent; procedures were approved by Oklahoma State University’s Institutional Review Board (Stillwater, OK, USA). Raw survey responses were stored securely within the Typeform survey platform (Typeform S.L., Barcelona, Spain), with access restricted to the two study investigators (J.B. and J.M.G.). Survey responses were not linked to participants’ SuppCo account records, and the dataset included no email address or SuppCo account identifier.

## 3. Results

### 3.1. Participant Characteristics

The analytic sample comprised 2094 current or recent creatine users with a reported duration of use (1055 women; 1039 men; 50.4% women) with a mean age of 53 years (SD: 12; women 54.0 [SD: 11.0], men 52.5 [SD: 12.6]). Median BMI was 25.1 kg/m^2^ (IQR: 21.9–29.8) among women and 27.1 kg/m^2^ (IQR: 24.8–31.4) among men, and the median daily dose was 5 g/day in both groups. Among the full eligible binary-gender sample (*n* = 2178, which includes the 84 cycling respondents), most respondents reported strength training (women 82.7%, men 86.7%) and general fitness or cardio (64.8% vs. 62.7%) as physical-activity types, with endurance and team sports more common among men and very few respondents of either gender reporting no physical activity (Supplementary Table S7).

Eighty-four users reported cycling creatine on and off without a placeable total duration (26.2% women; mean age 44.8 years; mean BMI 29.8 kg/m^2^) and were therefore held out of the duration-ordered analyses. Compared with the duration-placeable sample, cycling users were less often women (26.2% vs. 50.4%) and younger on average (44.8 vs. 53.0 years). Women comprised 49.4% of eligible (current and recent) binary-gender users when this group was included (*n* = 2178) and 50.4% of the duration-placeable sample (*n* = 2094).

### 3.2. Representation of Women Across Duration of Use

**Figure 1:**
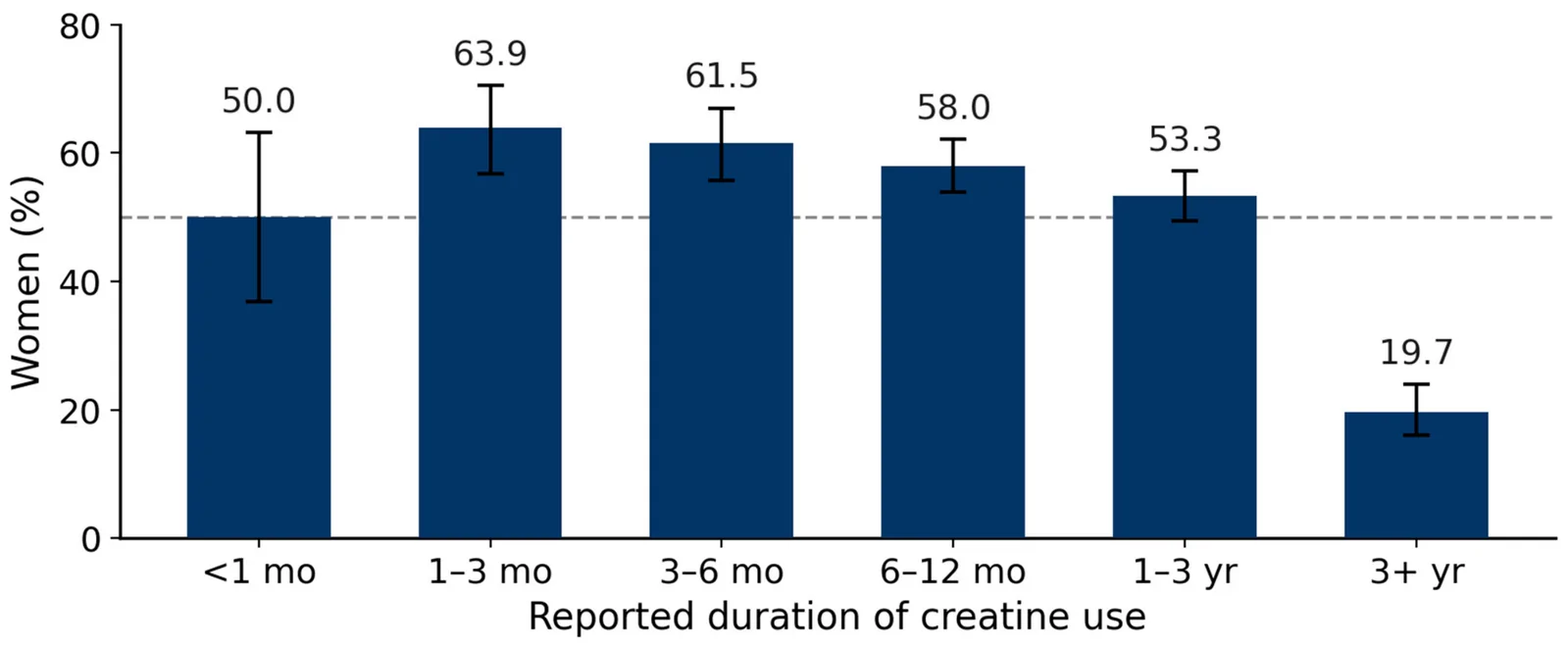
Share of creatine users who were women across the six ordered duration bands, with 95% Wilson confidence intervals. The dashed line marks 50%, indicating equal representation of women and men. mo, months; yr, years.

Women comprised 50.0% to 63.9% of respondents across the five duration categories below three years, compared with 19.7% in the 3+ year category (Table 1; Figure 1). The share of women was 50.0% at less than one month (*n* = 52), 63.9% at 1 to 3 months, then 61.5%, 58.0%, and 53.3% through 1 to 3 years, before falling to 19.7% at 3+ years. In the age-adjusted categorical model, duration was strongly associated with gender composition (global likelihood-ratio test *df* = 5, *p* < 0.001; *n* = 2055). Relative to 3+ years, the odds of being a woman were elevated in every shorter band, from 3.90 (95% CI 2.12 to 7.16) at under one month to 7.49 (5.04 to 11.24) at 1 to 3 months, with no ordering consistent with a constant per-band effect.

The pattern was robust to age adjustment. A Cochran–Armitage trend test across the ordered bands was significant (χ^2^ = 100.27, *df* = 1, *p* < 0.001), spline adjustment for age produced materially identical estimates, and the direction was consistent within the three age strata with adequate cell counts (30–49, 50–64, and 65–79 years); the 18–29 and 80+ strata were too sparse for reliable comparison (Supplementary Table S2).

### 3.3. Characteristics of Women Users Across Duration of Use

This section reports women only; gender comparisons appear in Section 3.4.

**Figure 2:**
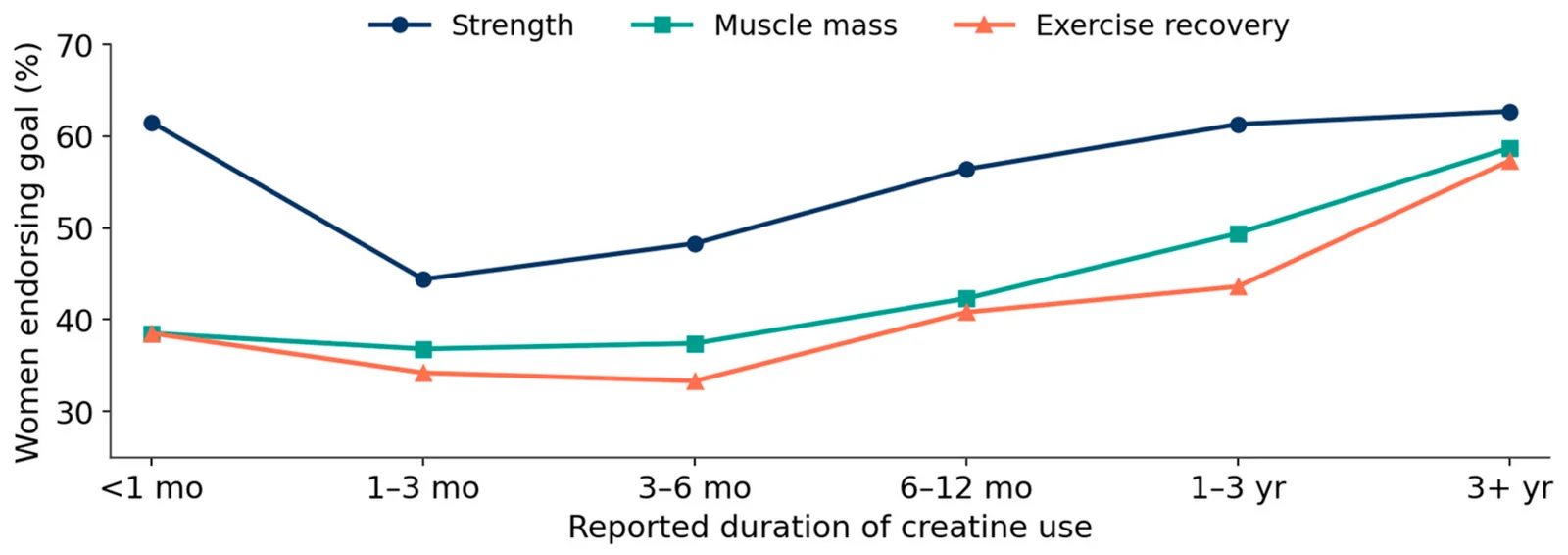
Endorsement of the three goals that differed across duration bands among women (strength, muscle mass, and exercise recovery), shown across all six ordered bands (Abbreviations: mo, months; yr, years).

Goals. Three of nine goals differed across the six duration bands after correction: strength, muscle mass, and exercise recovery (omnibus q = 0.022 each), each most endorsed at 3+ years (Table 2; Figure 2). At 3+ years, 62.7% of women endorsed strength, 58.7% muscle mass, and 57.3% exercise recovery (Table 2; Figure 2). Muscle-mass and exercise-recovery endorsement were highest in the 3+ year category, although their crude trajectories were not strictly monotonic; strength was likewise non-monotonic, endorsed by 61.5% of women at under one month, falling to 44.4% at 1 to 3 months and rising thereafter. Athletic performance also rose to 48.0% at 3+ years but did not survive correction (q = 0.112), nor did healthy aging (q = 0.106), which peaked at 6 to 12 months. Cognition, energy or fatigue, weight management, and general wellness did not differ significantly across bands.

Knowledge and myth beliefs. Mean knowledge alignment among women increased from 3.54 of 5 at under one month to 4.69 at 3+ years (Jonckheere–Terpstra z = 6.01, *p* < 0.001; Spearman rho = 0.185). The increase reflects declining uncertainty rather than declining error: mean “unsure” responses fell from 1.38 to 0.31 across bands, while mean incorrect responses remained essentially flat (0.08 to 0.00). Active myth endorsement was rare throughout, and no individual belief item differed across duration categories after correction (Supplementary Table S8; all q ≥ 0.102). Shorter-duration women scored lower on knowledge alignment but did not actively endorse more misconceptions; rather, the lower score reflects a higher share of ”unsure” responses. In other words, women in shorter-duration categories appeared more uncertain, not more misinformed.

Information sources. One of seven sources differed across bands after correction: Coach-or-trainer endorsement rose with duration, from 9.4% at 1 to 3 months to 29.3% at 3+ years (q = 0.002). Friend-or-family endorsement was highest at under one month and generally lower in later categories (46.2% at under one month, 24.8% at 1 to 3 months, 21.3% at 3+ years) but did not survive correction (q = 0.052). Podcast endorsement peaked in the 1 to 3 year band (38.1%) but also did not survive correction (q = 0.058). Social media, YouTube, healthcare provider, and research articles did not differ significantly across bands.

**Covariate-adjusted sensitivity analyses.** Mean physical-activity level was 3.32 among women and 3.36 among men on the 1–5 scale and differed across duration categories among women, ranging from 3.00 in the 1–3-month category to 3.65 at 3+ years (Jonckheere–Terpstra *p* < 0.001). Because age, physical activity, BMI, and duration were measured concurrently, covariate-adjusted models are presented as sensitivity analyses. Among women with complete covariate data (*n* = 1016), none of the nine goal outcomes showed a significant global duration effect after adjustment (all q ≥ 0.344; Supplementary Table S9); the largest global test statistic was observed for muscle-mass endorsement (χ^2^ = 8.97, *df* = 5; q = 0.344). Coach-or-trainer endorsement remained associated with duration in the adjusted categorical model (χ^2^ = 21.15, *df* = 5; q = 0.005), with higher endorsement in the longer-duration categories. Friend-or-family endorsement showed a nominal inverse pattern, with lower adjusted odds in each longer-duration category relative to the <1-month category, but the global association did not survive correction in either the unadjusted omnibus analysis (q = 0.052) or the adjusted categorical model (χ^2^ = 13.01, *df* = 5; q = 0.082). Knowledge alignment remained associated with duration after adjustment (global categorical F-test: F[5, 1007] = 7.76; *p* < 0.001). Adding strength-training participation as a covariate to the strength, muscle-mass, exercise-recovery, and athletic-performance models did not change these conclusions.

### 3.4. Gender Comparisons

**Figure 3:**
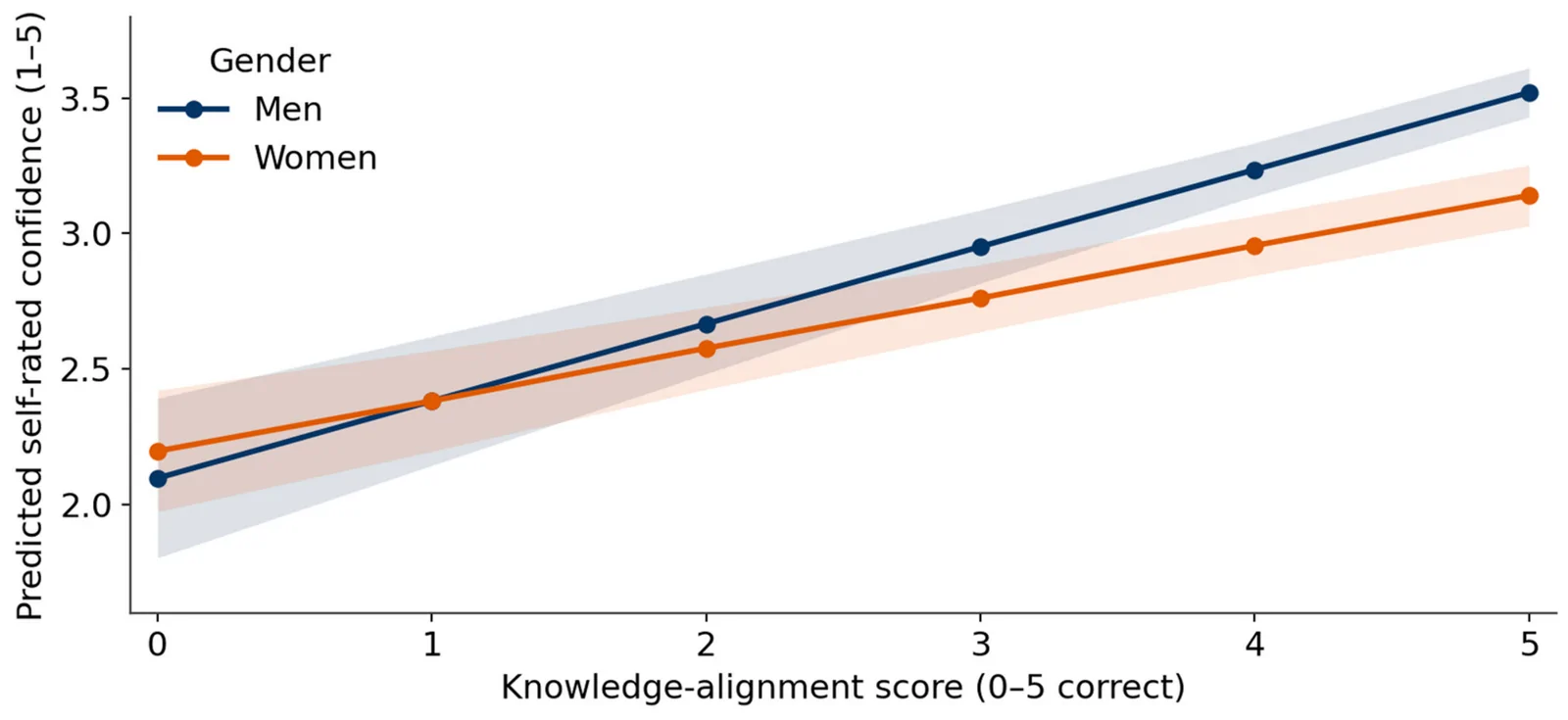
Self-rated confidence as a function of knowledge alignment, by gender, with 95% confidence intervals (*n* = 2055). Men’s confidence rises more steeply with knowledge than women’s (gender × knowledge interaction), so the gap is largest at the highest knowledge-alignment scores. Shaded bands represent 95% confidence intervals around the model-predicted means.

**Confidence.** Self-rated confidence increased with knowledge alignment, and the gender difference was not constant across the knowledge range (gender × knowledge interaction β = −0.096, 95% CI −0.171 to −0.022, *p* = 0.012; *n* = 2055; Table 3; Figure 3). Confidence rose more steeply with knowledge among men (0.284 points per item) than among women (0.188 points per item). The women–men difference was not distinguishable from zero at low knowledge-alignment scores and emerged and widened across the upper range: −0.19 points (95% CI −0.32 to −0.06) at a score of 3, −0.28 (−0.37 to −0.20) at 4, and −0.38 (−0.47 to −0.29) at 5, where 67% of the sample sits. The women–men difference in self-rated confidence was therefore largest among respondents with the highest observed score on the five-item measure. The 0.38-point difference is modest on the 1–5 confidence scale and is interpreted as a descriptive difference rather than a clinically meaningful threshold.

**Goals and information sources.** In age- and duration-adjusted models, women were less likely than men to endorse athletic performance as a goal (OR 0.58, 95% CI 0.48 to 0.70, q < 0.001) and more likely to endorse healthy aging (OR 1.41, 1.16 to 1.71, q = 0.002); no other goal differed after correction. Six of seven information sources differed: women were more likely to report podcasts (OR 1.67, 1.36 to 2.05), healthcare providers (1.74, 1.24 to 2.45), and social media (1.35, 1.08 to 1.68), and less likely to report YouTube (0.38, 0.27 to 0.52), research articles (0.66, 0.54 to 0.80), and friends or family (0.78, 0.62 to 0.97); all q ≤ 0.029. Coach or trainer did not differ (q = 0.127). Women also scored lower on knowledge alignment (β = −0.278, 95% CI −0.371 to −0.186, *p* < 0.001). Full results for all nine goals, seven information sources, and the knowledge-alignment score appear in Supplementary Table S4. No gender × duration-group interaction survived correction for any of the 16 items (all q ≥ 0.830; Supplementary Table S5). Probability-scale estimates were consistent with the odds-ratio results. The largest adjusted difference among goals was for athletic performance (37.5% among women vs. 50.2% among men; adjusted prevalence difference −12.7 percentage points). Among information sources, the largest differences were for podcasts (+9.8 points), research articles (−9.0 points), and YouTube (−8.3 points). Differences for healthy aging (+7.9 points) and healthcare-provider guidance (+4.2 points) were smaller in absolute terms despite relatively large odds ratios (Supplementary Table S4).

**Figure 4:**
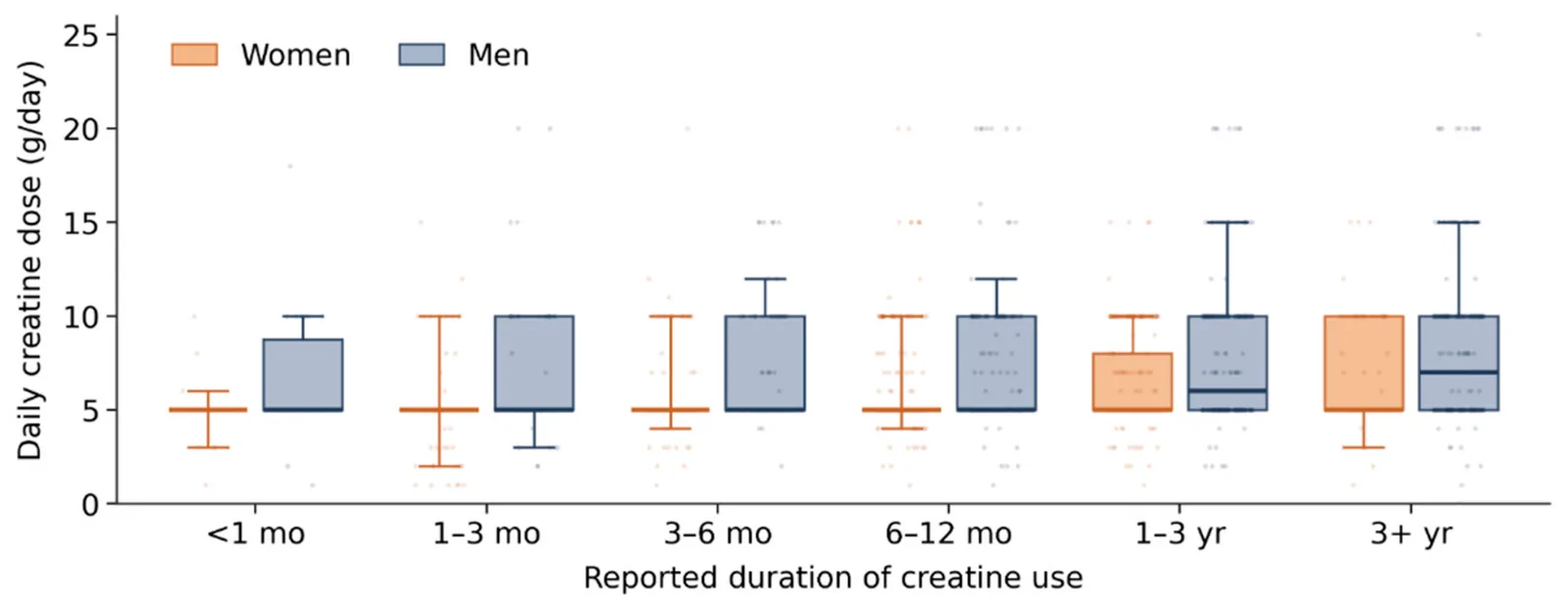
Daily creatine dose by duration band and gender, shown as box plots (median and interquartile range; whiskers extend to 1.5 × IQR). Individual points are jittered raw observations for each respondent. Doses > 30 g/day were set to missing. Abbreviations: mo, months; yr, years.

**Supplementation behavior.** Median daily dose was 5 g/day in both genders, but women clustered more tightly on that value: 69.3% reported exactly 5 g/day versus 50.9% of men, and the interquartile range among women spanned 5 to 5 g/day through the 6 to 12 month band while the range among men reached 10 g/day from the 1 to 3 month band onward (Figure 4; Supplementary Table S3).

Prior loading-phase use was more common among men (20.3% versus 12.3%), whereas women more frequently selected the response option “not sure what a loading phase is” (13.0% versus 6.3%). Selection of that option declined with duration in both genders, from 15.4% and 42.3% at under one month (*n* = 26 in each gender) to 8.0% and 1.0% at 3+ years among women and men respectively.

**Satisfaction and continuation.** Satisfaction was high and six-month continuation intent near ceiling in both genders across all bands, with respondents who did not select “yes” to intended six-month continuation concentrated among the shortest-duration users (16 women and 4 men in the ≤3-month band, of 49 such respondents overall; Supplementary Table S6).

Sensitivity analyses supported the primary findings. The gender × knowledge-alignment confidence interaction was materially unchanged with HC3-robust inference and an ordinal confidence model, and the women–men difference in knowledge alignment was concordant across linear, ordinal, quasi-binomial, Rasch, and 2PL specifications (Supplementary Table S10).

## 4. Discussion

This study characterizes women who use creatine during a period in which creatine is increasingly discussed beyond sports performance and within broader women’s-health and healthy-aging contexts. Rather than evaluating creatine efficacy, this analysis examined whether women using creatine in this sample align with the populations, outcomes, information pathways, and assumptions that have historically shaped the evidence base. The findings describe a cross-sectional pattern that differs by reported duration of use. Relative to men, women were concentrated at shorter durations of use: they were a majority or near-majority of users in every band under three years and a clear minority at three or more years. They also endorsed healthy-aging goals more often and athletic-performance goals less often than men, reached creatine through different information channels, and reported lower knowledge alignment, with the gender difference in self-rated confidence emerging at higher knowledge alignment. Together, these patterns suggest that the women in this sample are using creatine for different reasons and through different information channels than the largely performance-focused populations of men that generated much of the current evidence base. This divergence between the observed pattern of use and the available evidence has potential implications for research priorities and consumer education.

### 4.1. How Women in This Sample Use Creatine

Creatine has traditionally been centered within sports nutrition, with research primarily focused on strength, power, lean mass, and athletic performance [1,2]. Over the past several years, discussion of creatine has expanded into women’s health, menopause, cognition, healthy aging, fatigue, and brain health. Recent reviews have proposed a lifespan framework for creatine supplementation in women, highlighting potential relevance across menstruation, pregnancy, perimenopause, and menopause, while acknowledging gaps in the evidence base [3,4,5].

The goals reported in this sample are consistent with this broader interest in creatine beyond athletic performance. Women comprised 63.9% of those using creatine for one to three months but only 19.7% of those reporting three or more years of use. This pattern was robust to age adjustment and held within three age strata with adequate cell counts. Because these data are cross-sectional, capturing current or recent users at a single point in time, the pattern cannot be interpreted causally. Women may have begun using creatine only recently, or may discontinue at higher rates than men; these explanations cannot be distinguished here and carry different educational implications: if the pattern reflects more recent initiation of use by women, the priority is education and expectation-setting at the start of use; if it reflects lower long-term persistence among women, the priority is prospective study of why women continue or stop. Neither can be inferred from these cross-sectional data.

The goal mix among women also differed across duration, though not in a simple gradient. Strength, muscle mass, and exercise recovery all peaked among the longest-duration users, while cognition, healthy aging, energy, and general wellness showed no significant differences across bands. The gender contrast is more informative: pooled across duration groups, women were substantially less likely than men to cite athletic performance and substantially more likely to cite healthy aging.

Taken together, the profile that emerges is a midlife woman for whom healthy aging is a more prominent motivation than athletic performance; cognition was commonly endorsed by both women and men and did not distinguish the genders after correction. Because menopausal status was not assessed, reproductive stage is not inferred from age, and whether this profile corresponds to the peri- and postmenopausal applications emphasized in the current women’s-health creatine literature [3,4,5,6,11] cannot be determined here. Women in this sample may therefore be using creatine for different reasons and through different information pathways than the populations that generated much of the existing evidence base. This misalignment has translational importance: the scientific questions driving current interest in creatine among women no longer align fully with the outcomes that have historically dominated creatine research.

### 4.2. The Evidence-to-Consumer Gap

Interest in creatine for cognition, healthy aging, menopause, and women’s health has grown substantially, but the intervention data for these indications remain far more limited and heterogeneous than for traditional performance applications. The most recent systematic review of creatine supplementation in active women found mixed performance outcomes [7]; the largest postmenopausal trial to date was null on its primary bone-density endpoint [8]; and although pooled analyses suggest a modest lean-mass benefit in women [9], strength benefits in older women have been more evident in studies lasting 24 or more weeks that combined supplementation with resistance training [10]. The cognitive and healthy-aging benefits women most commonly seek are biologically plausible and under active investigation [5,15,16], but onset timelines, optimal dosing, and effect sizes in peri- and postmenopausal populations remain incompletely characterized.

Many women in the present sample reported shorter total durations of use and were motivated by outcomes reaching beyond musculoskeletal performance, in areas where women-specific trial evidence is least mature. This immaturity is in part structural. Women have historically been underrepresented as participants in sport, exercise, and nutrition research, comprising roughly a third of participants across major journals in recent years, with only a small minority of studies conducted exclusively in women [17,18]. When women are enrolled, they are frequently studied in small mixed-sex subgroups, and hormonal status, menopausal stage, and menstrual phase are seldom characterized.

This pattern illustrates how consumer interest can extend into areas where the clinical evidence base remains limited and heterogeneous, particularly when emerging findings are amplified through social media, podcasts, peer networks, and commercial wellness channels. Understanding who is using creatine, what benefits they expect, and where they source their information is therefore an essential complement to efficacy research.

### 4.3. Knowledge, Confidence, and Implementation

These patterns have practical implications for education. Educational efforts often focus on correcting misconceptions or adding facts, but the present findings suggest that shorter-duration women users are characterized less by active misconceptions than by unresolved uncertainty. Mean “unsure” responses on the knowledge battery fell steadily across duration groups among women, while mean incorrect responses stayed flat and low.

The confidence findings add a second, distinct pattern. At higher knowledge-alignment scores, women rated their understanding lower than men; the difference was not uniform: it was indistinguishable from zero among the small number of respondents with low knowledge-alignment scores and widened steadily across the upper range, reaching roughly four tenths of a point at the maximum score, where two thirds of the sample sits. The difference was largest among respondents with the highest observed score. This pattern is consistent with a well-documented gender gap in self-assessed confidence that persists even at equivalent objective performance and that tends to be taken at face value rather than adjusted for a tendency to understate [19].

Information-source patterns were heterogeneous rather than uniformly shifting with duration. Among women, friend-or-family endorsement was highest at under one month and generally lower in subsequent categories, whereas coach-or-trainer endorsement rose with duration. Compared with men, women were more likely to report podcasts, healthcare providers, and social media, and less likely to report YouTube, research articles, and friends or family. Several of these are informal or consumer-facing channels rather than clinical or primary-scientific ones. These findings identify the channels through which women encounter creatine but do not establish the quality, content, or influence of the information encountered through them.

Timing is potentially relevant because creatine’s perceived time to benefit may differ by outcome. Muscle creatine stores can saturate within approximately one month at standard doses, a timeline that likely shapes popular expectations of how quickly creatine works. In contrast, outcomes particularly relevant to older women, such as meaningful gains in strength, have been more evident in studies lasting 24 or more weeks that combined consistent use with resistance training [10]. Whether users’ expectations about time to benefit align with these longer-term outcomes was not assessed here. Whether some users discontinue before longer-term benefits would be expected to emerge cannot be determined from the present cross-sectional data, which include current and recent past-12-month users but not longer-term discontinuers; this is raised as a hypothesis for future longitudinal research rather than as a finding of this study.

Educational efforts may therefore need to address uncertainty and confidence in addition to factual misconceptions. Educational content in the channels where these users actually encounter creatine, including platform material, product labeling, clinician talking points, and podcast-facing explainers, may also need to help users calibrate their understanding, set realistic expectations, and interpret uncertainty, especially where the science is still developing.

### 4.4. Implications for Future Research

Recent publications have emphasized the need for more women-specific creatine research [3,4]. The present findings suggest that future work should investigate not only physiological efficacy but also the population of women represented across duration categories in this sample. Several of the motivations most commonly reported in this cohort, including cognition, healthy aging, fatigue, and wellness, correspond to areas in which clinical evidence remains comparatively limited or heterogeneous.

Three priorities follow. First, trials should align their endpoints more closely with the reasons women actually choose creatine; healthy aging and cognition were among the most common motivations here, and healthy aging was the one goal women endorsed significantly more often than men, yet much of the existing literature still centers on performance, body composition, and bone outcomes. Second, the knowledge and confidence gaps documented here are potentially modifiable targets for educational and implementation research, and the finding that the confidence gap is widest among women with the highest knowledge-alignment scores suggests that knowledge alignment alone does not explain the observed confidence difference. Third, prospective cohort studies are needed to determine whether the observed cross-sectional duration distribution reflects differences in initiation timing, persistence, or other cohort and platform processes, and to identify the factors that shape long-term use among women.

More broadly, research on creatine in women may benefit from moving past whether creatine works and toward who is using it, why, and whether those motivations align with the populations and outcomes currently represented in the evidence base. As creatine is increasingly discussed in women’s-health and healthy-aging contexts, understanding this user population may matter increasingly for researchers and clinicians alike.

### 4.5. Limitations

Several limitations qualify these findings. The sample was recruited from a supplement-tracking application, which biases toward engaged consumers, is subject to nonresponse bias given the approximately 2% response rate, and limits generalizability to women using creatine outside such platforms.

The duration item asked respondents how long they had used creatine “in total” and therefore does not establish uninterrupted supplementation, calendar time of initiation, adherence, persistence, or retention. Accordingly, all duration-based findings are interpreted only as cross-sectional differences according to respondents’ reported total duration of creatine use. The observed cross-sectional duration distribution is therefore equally consistent with differences in initiation timing, differential persistence, platform-participation effects, recruitment dynamics, duration misclassification, or any combination of these. Because demographic data for the full invited population were unavailable, the direction and magnitude of nonresponse and selection bias cannot be quantified, and all duration comparisons among current and recent users are subject to survivor and selection processes that cannot separate duration of use from cohort or platform-tenure effects. All dose, duration, BMI, knowledge, confidence, and outcome measures are self-reported, and gender was self-reported rather than derived from biological sex, which should be considered when interpreting the specific physiology that motivates this work. The five-item knowledge-alignment score is a brief index rather than a validated instrument, and its content, criterion, and construct validity and measurement invariance across gender were not established. The survey did not capture pregnancy or lactation status, menopausal stage, menstrual status, detailed training and nutrition variables, protein or dietary creatine intake, renal disease, clinician advice, health literacy, exposure to specific media claims, or socioeconomic, educational, geographic, and racial or ethnic characteristics.

Benjamini–Hochberg correction was applied within each family of item-level tests but not across families; analyses outside the primary representation analysis should therefore be interpreted as exploratory. In particular, although strength, muscle-mass, and exercise-recovery goals differed across duration categories in the unadjusted within-women analyses, these associations were not significant after adjustment for age, physical activity, and BMI and therefore should not be interpreted as independent duration effects. In contrast, associations of duration with knowledge alignment and coach-or-trainer endorsement remained statistically significant after adjustment, whereas the friend-or-family association did not survive multiplicity correction. Gender × duration interaction analyses were exploratory, and null interaction results should not be interpreted as evidence of equivalence. Continuation intent is a stated attitude rather than observed retention. Because the sample comprises current and recent (past-12-month) users, it partially captures recent discontinuers but not longer-term ones, so satisfaction and continuation patterns remain subject to survivorship and are reported descriptively. Finally, the cohort is overwhelmingly midlife and older (mean age 53 years); findings should not be extrapolated to younger active women, for whom menstrual-cycle-aware work applies more directly [20,21].

## 5. Conclusions

Among current and recent SuppCo users who responded to this survey, women comprised a larger share of respondents in shorter reported duration-of-use categories than in the 3+ year category and differed from men in several goals, information-source endorsements, knowledge alignment, and the relationship between knowledge alignment and self-rated confidence. Women more often endorsed healthy aging and less often endorsed athletic performance, while the confidence difference between women and men widened at higher knowledge-alignment scores. These findings are specific to this platform-based convenience sample and should not be interpreted as representative of women using creatine more broadly. The patterns observed here extend beyond the performance-focused populations and outcomes that have historically dominated the creatine evidence base. As creatine is increasingly discussed in women’s-health and healthy-aging contexts, these findings highlight the need for women-focused research that addresses not only efficacy, but also the information pathways, knowledge, confidence, and goals that shape how women engage with creatine supplementation.

## Figures and Tables

**Table 1:** Share of creatine users who were women, across the six ordered duration bands.

Duration band	Total *n*	Women *n*	Women %	95% CI (Wilson)
<1 month	52	26	50.0	36.9–63.1
1–3 months	183	117	63.9	56.8–70.5
3–6 months	283	174	61.5	55.7–67.0
6–12 months	550	319	58.0	53.8–62.1
1–3 years	645	344	53.3	49.5–57.2
3+ years	381	75	19.7	16.0–24.0

Note. Age-adjusted categorical logistic model, global likelihood-ratio test *df* = 5, *p* < 0.001 (*n* = 2055); odds of being a woman relative to the 3+ year band were 3.90 (95% CI 2.12–7.16) at <1 month, 7.49 (5.04–11.24) at 1–3 months, 6.42 (4.53–9.18) at 3–6 months, 5.57 (4.11–7.62) at 6–12 months, and 4.69 (3.49–6.37) at 1–3 years. Cochran–Armitage trend test χ^2^ = 100.27, *df* = 1, *p* < 0.001. Wilson intervals computed from per-band totals. Age-stratified counts appear in Supplementary Table S2.

**Table 2:** Endorsement across duration bands among women (%): goals, information sources, and creatine knowledge.

Item	<1 mo	1–3 mo	3–6 mo	6–12 mo	1–3 yr	3+ yr	*p*/q (BH)
*Goals (% endorsing)*							
Strength	61.5	44.4	48.3	56.4	61.3	62.7	0.022
Muscle mass	38.5	36.8	37.4	42.3	49.4	58.7	0.022
Exercise recovery	38.5	34.2	33.3	40.8	43.6	57.3	0.022
Athletic performance	30.8	29.1	29.3	36.4	36.0	48.0	0.112
Healthy aging/longevity	53.8	65.0	61.5	72.4	70.6	62.7	0.106
Cognition	57.7	66.7	62.6	70.5	70.9	61.3	0.240
Weight management	23.1	15.4	13.8	10.3	9.3	13.3	0.217
General wellness	34.6	45.3	39.1	35.7	40.1	48.0	0.332
Energy/fatigue	46.2	40.2	36.2	33.9	32.0	33.3	0.478
*Information sources (% reporting)*							
Coach or trainer	7.7	9.4	9.2	17.6	17.7	29.3	0.002
Friend or family	46.2	24.8	21.3	19.4	17.4	21.3	0.052
Podcasts	23.1	23.1	33.9	35.1	38.1	25.3	0.058
Social media	23.1	29.1	25.9	32.9	23.0	20.0	0.092
Healthcare provider	3.8	14.5	9.8	12.5	11.0	5.3	0.390
YouTube	0.0	7.7	7.5	6.6	4.9	8.0	0.546
Research articles	15.4	27.4	24.1	25.4	27.9	33.3	0.546
*Knowledge (mean of 0–5)*							
Knowledge-alignment score	3.54	3.98	3.95	4.14	4.39	4.69	**<0.001 †**
Mean “unsure” responses	1.38	0.95	0.98	0.77	0.57	0.31	—
Mean incorrect responses	0.08	0.07	0.07	0.09	0.05	0.00	—

Note. *n* per band (women) = 26, 117, 174, 319, 344, and 75. Binary items were tested with an order-free omnibus test of homogeneity across the six bands (Monte Carlo *p*, 10,000 replicates, where expected cell counts fell below 5), with Benjamini–Hochberg correction within domain (9 goals; 7 sources, SuppCo excluded as the recruitment platform and therefore not comparable to the other sources). The knowledge-alignment score was tested with the Jonckheere–Terpstra ordered-alternatives test (z = 6.01); its value in the final column (marked †) is an unadjusted *p*-value and is not part of any Benjamini–Hochberg-corrected family, whereas every other value in that column is a BH-adjusted q-value. Bold indicates q < 0.05 (goal and information-source items only). Abbreviations: mo, months; yr, years.

**Table 3:** Self-rated confidence as a function of knowledge alignment and gender. **Panel A.** Knowledge slopes and gender interaction. **Panel B.** Women–men difference in confidence at each knowledge-alignment score.

**(A)**		
**Predictor**	**β (95% CI)**	***p***
Knowledge-alignment score, men (points per item)	0.284 (0.224, 0.345)	<0.001
Women vs. men at knowledge-alignment score 5 (points)	−0.381 (−0.469, −0.292)	<0.001
Knowledge-alignment score, women, derived (points per item)	0.188 (0.142, 0.234)	—
Difference in slopes (knowledge × woman)	−0.096 (−0.171, −0.022)	0.012
**(B)**		
**Knowledge-Alignment Score**	**Difference**	**95% CI**
0	+0.10	−0.24, 0.44
1	+0.00	−0.26, 0.27
2	−0.09	−0.29, 0.11
3	−0.19	−0.32, −0.06
4	−0.28	−0.37, −0.20
5	−0.38	−0.47, −0.29

Note. Outcome: self-rated confidence (1–5). Model: confidence ~ knowledge × woman + duration + age; *n* = 2055. The women term is the gender difference at a knowledge-alignment score of 5, where 67% of the sample sits; the difference at any other score k equals this term plus (k − 5) × the interaction. Because the difference varies with knowledge, Panel B reports it at each observed score. The slope among women is the sum of the slope among men and the interaction; its confidence interval accounts for the covariance between them.

## Data Availability

The study dataset is owned by SuppCo. De-identified survey data supporting the findings of this study are available from the corresponding author upon reasonable request, subject to SuppCo’s data-sharing policies.

## Supplementary Materials

The following supporting information can be downloaded at https://www.mdpi.com/article/10.3390/nu18182960/s1. Table S1: Per-model analytic sample sizes, with a variable-specific missing-data panel. Table S2: Age-stratified representation across all six ordered duration bands. Table S3: Dosing behavior by duration band and gender. Table S4: Pooled gender comparisons across all modelled outcomes, with odds ratios and adjusted prevalences, adjusted prevalence differences, and prevalence ratios. Table S5: Gender × duration interaction tests. Table S6: Satisfaction and six-month continuation intent by gender. Table S7: Baseline characteristics, supplementation behaviors, and physical-activity level by gender and duration band, with physical-activity types tabulated by gender. Table S8: Per-item belief (myth) endorsement among women across duration. Table S9: Age-, activity-, and BMI-adjusted within-women categorical duration effects. Table S10: Sensitivity analyses for the confidence and knowledge-alignment measures (confidence-model robustness, bounded knowledge-score models, and item-response-theory scoring). Figure S1: participant flow. Supplementary File S1: The administered questionnaire and complete codebook (item wording, response options and permitted selections, conditional branching, coding, and scoring rules).

## Abbreviations

ATP: adenosine triphosphate; BH, Benjamini–Hochberg; BMI, body mass index; CI, confidence interval; HC3, heteroscedasticity-consistent standard errors; IQR, interquartile range; IRB, institutional review board; IRT, item-response theory; KR-20, Kuder–Richardson formula 20; LRT, likelihood-ratio test; OR, odds ratio; PD, adjusted prevalence difference; PO, proportional odds; PR, prevalence ratio; SD, standard deviation.
