# Supplementary material for: Creatine Use Among Women Across Reported Duration-of-Use Groups: A Platform-Based Cross-Sectional Survey of Supplementation Behaviors, Motivations, Knowledge, and Experiences
Source: Nutrients. 2026 Sep 9;18(18):2960. doi: 10.3390/nu18182960 (PMC13610488; doi:10.3390/nu18182960)
Supplement: Supplementary file 1 [file nutrients-18-02960-s001.zip › nutrients-4496635-supplementary.pdf]

nutrients MDPI

Supplementary Materials

Table S1. Per-model analytic sample sizes.

Analysis	N	Complete-case requirement
Primary analytic sample	2,094	current or past-12-month use; placeable reported duration
Representation, age-adjusted	2,055	+ age
Pooled gender comparisons	2,055	+ age
Confidence model	2,055	+ confidence; knowledge; age
Within-women trends (unadjusted)	1,055	women; duration
Within-women trends (adjusted)	1,016	women; + age; activity; BMI
Dose-specific analyses	2,087	+ valid daily dose (≤ 30 g/day)
Bounded knowledge-score sensitivity (Table S10, Panel B)	1,934	+ duration; age; activity; BMI
IRT scoring sensitivity (Table S10, Panel C)	2,139	+ age; knowledge items

Note. The primary analytic sample (2,094) is constructed once; each model applies further complete-case filters on only the variables it uses. Relative to 2,094, the age-adjusted models exclude 39 respondents missing age; goal and information-source endorsements are non-missing by construction (checkbox items). Duration-based analyses begin from the 2,094 primary sample; the item-response-theory scoring sensitivity did not require a placeable duration and therefore used a larger sample (n = 2,139).

Table S1, Panel B. Variable-specific missing data (primary analytic sample, n = 2,094).

Variable	Missing n	Available n	% missing
Age	39	2,055	1.9
BMI	124	1,970	5.9
Physical-activity level	0	2,094	0.0
Self-rated confidence	0	2,094	0.0
Knowledge-alignment score	0	2,094	0.0
Daily dose (analysis)	7	2,087	0.3
Dosing frequency	0	2,094	0.0
Loading-phase use	0	2,094	0.0

Note. Missingness computed on the primary analytic sample (n = 2,094). Physical-activity level, self-rated confidence, knowledge-alignment score, dosing frequency, and loading-phase use were complete. Analysis dose was set to missing only for the seven respondents reporting more than 30 g/day; these respondents were retained in all non-dose analyses.

Table S2. Age-stratified representation of women across all six ordered duration bands.

Age stratum	Duration band	n	Women n	Women %
18–29	<1 mo	2	1	50.0
	1–3 mo	6	5	83.3
	3–6 mo	8	4	50.0
	6–12 mo	16	3	18.8
	1–3 yr	17	7	41.2

Age stratum	Duration band	n	Women n	Women %
	3+ yr	22	4	18.2
30–49	<1 mo	19	9	47.4
	1–3 mo	53	36	67.9
	3–6 mo	88	57	64.8
	6–12 mo	165	96	58.2
	1–3 yr	220	121	55.0
	3+ yr	175	29	16.6
50–64	<1 mo	18	8	44.4
	1–3 mo	87	57	65.5
	3–6 mo	117	71	60.7
	6–12 mo	255	166	65.1
	1–3 yr	304	169	55.6
	3+ yr	136	26	19.1
65–79	<1 mo	12	7	58.3
	1–3 mo	33	19	57.6
	3–6 mo	64	38	59.4
	6–12 mo	104	48	46.2
	1–3 yr	88	41	46.6
	3+ yr	44	15	34.1
80+	<1 mo	0	0	0.0
	1–3 mo	1	0	0.0
	3–6 mo	0	0	0.0
	6–12 mo	0	0	0.0
	1–3 yr	1	0	0.0
	3+ yr	0	0	0.0

Note. Age strata are in years. Cells give the count and women's share within each age × duration combination (cycling users excluded). Sparse cells (n < 5) are shown for completeness. The direction of the representation difference was consistent within the 30–49, 50–64, and 65–79 strata. The 18–29 and 80+ strata contained too few respondents to support the comparison. Abbreviations: mo, months; yr, years.

Table S3. Dosing behavior by duration band and gender.

Duration band	Gender	n	Median (Q1–Q3)	Mean (g/day)	% exactly 5 g/day	% once daily
<1 mo	Women	26	5 (5–5)	4.88	69.2	88.5
	Men	26	5 (5–8.75)	6.38	65.4	73.1
1–3 mo	Women	117	5 (5–5)	5.10	71.8	79.5
	Men	66	5 (5–10)	6.74	57.6	71.2
3–6 mo	Women	172	5 (5–5)	5.61	78.5	84.5
	Men	109	5 (5–10)	6.85	61.5	77.1

Duration band	Gender	n	Median (Q1–Q3)	Mean (g/day)	% exactly 5 g/day	% once daily
6–12 mo	Women	315	5 (5–5)	5.91	73.7	85.6
	Men	231	5 (5–10)	7.05	58.4	79.2
1–3 yr	Women	344	5 (5–8)	6.31	63.1	81.7
	Men	300	6 (5–10)	7.82	47.0	77.7
3+ yr	Women	75	5 (5–10)	6.68	54.7	89.3
	Men	306	7 (5–10)	8.08	42.5	69.9

Note. Dose in g/day. Dose values above 30 g/day were set to missing for dose-specific analyses (n = 7; all > 250 g/day); these respondents were retained in all non-dose analyses. Dose statistics use the dose-valid subset (the n shown per row); the percentage taking creatine once daily is computed on the frequency-valid sample, which was complete for all 2,094 respondents, including the seven with implausible dose. Once-daily and multiple-times-daily were the two predominant frequencies (1,665 and 268 respondents respectively); a few times per week (114), inconsistently (25), and only during training phases (22) accounted for the balance. Women clustered on 5 g/day across duration of use. Abbreviations: mo, months; yr, years.

Table S4. Pooled gender comparisons across all modelled outcomes, with odds ratios and adjusted probability-scale estimates.

Outcome	OR (95% CI)	q	% women	% men	PD, pp	PR
**Goals (% endorsing)**						
Athletic performance	0.58 (0.48, 0.70)	<0.001	37.5	50.2	−12.7	0.75
Healthy aging / longevity	1.41 (1.16, 1.71)	0.002	67.5	59.6	+7.9	1.13
Exercise recovery	0.84 (0.70, 1.01)	0.179	43.4	47.4	−4.0	0.92
Energy / fatigue	0.84 (0.70, 1.02)	0.179	34.7	38.4	−3.7	0.90
Weight management	1.26 (0.93, 1.70)	0.241	11.2	9.1	+2.1	1.23
Muscle mass	0.88 (0.73, 1.05)	0.242	46.8	49.9	−3.2	0.94
Strength	0.90 (0.75, 1.09)	0.353	57.6	60.0	−2.4	0.96
General wellness	1.08 (0.90, 1.31)	0.452	40.5	38.6	+1.9	1.05
Cognition	1.00 (0.82, 1.21)	0.965	68.5	68.4	+0.1	1.00
**Information sources (% reporting)**						
YouTube	0.38 (0.27, 0.52)	<0.001	5.9	14.2	−8.3	0.41
Podcasts	1.67 (1.36, 2.05)	<0.001	31.8	22.0	+9.8	1.44
Research articles	0.66 (0.54, 0.80)	<0.001	27.5	36.6	−9.0	0.75
Healthcare provider	1.74 (1.24, 2.45)	0.002	10.4	6.2	+4.2	1.68
Social media	1.35 (1.08, 1.68)	0.010	25.1	19.9	+5.1	1.26
Friend or family	0.78 (0.62, 0.97)	0.029	20.3	24.5	−4.2	0.83
Coach or trainer	1.22 (0.95, 1.57)	0.127	17.5	14.9	+2.6	1.17
**Knowledge**						
Knowledge-alignment score (β)	−0.278 (−0.371, −0.186)	—	—	—	—	—

Note: Odds ratios (women vs men) are the age- and duration-adjusted model coefficients (outcome ~ woman + duration group + age); q-values are Benjamini–Hochberg-adjusted within the goal and information-source domains. Adjusted percentages, adjusted prevalence differences (PD, percentage points), and prevalence ratios (PR) are marginal standardized estimates from the same models, averaging predicted probabilities over the observed covariate distribution. The knowledge-alignment score is continuous (linear regression coefficient β); probability-scale columns do not apply. n = 2,055.

Table S5. Gender × duration interaction tests, duration as a four-level duration group.

Item	df	LRT χ^2	p	q (BH)
Social media	3	6.65	0.084	0.830
Weight management	3	6.17	0.104	0.830
Exercise recovery	3	5.07	0.166	0.888
Friend or family	3	4.26	0.235	0.899
Podcasts	3	3.69	0.297	0.899
YouTube	3	3.23	0.357	0.899
Healthy aging / longevity	3	2.61	0.456	0.899
Cognition	3	2.15	0.541	0.899
Athletic performance	3	2.12	0.547	0.899
Strength	3	2.02	0.568	0.899
Energy	3	1.79	0.618	0.899
General wellness	3	1.48	0.688	0.917
Coach or trainer	3	0.74	0.865	0.959
Research articles	3	0.70	0.873	0.959
Muscle mass	3	0.43	0.935	0.959
Healthcare provider	3	0.30	0.959	0.959

Note. Likelihood-ratio test for the gender × duration-group interaction block in models of the form outcome ~ duration group × woman + age (n = 2,055). Benjamini-Hochberg correction across the 16-item family.

Table S6. Satisfaction and six-month continuation intent by gender across duration bands.

Duration band	Gender	n	Satisfaction, mean (SD)	% intending to continue
<1 mo	Women	26	6.4 (2.1)	88.5
	Men	26	6.7 (1.7)	92.3
1–3 mo	Women	117	6.9 (2.0)	88.9
	Men	66	6.8 (2.1)	97.0
3–6 mo	Women	174	6.9 (2.1)	94.8
	Men	109	7.6 (1.7)	97.2
6–12 mo	Women	319	7.7 (1.9)	97.5
	Men	231	7.8 (1.8)	97.8
1–3 yr	Women	344	8.0 (1.6)	99.7
	Men	301	8.2 (1.6)	99.7
3+ yr	Women	75	8.6 (1.7)	100.0

Duration band	Gender	n	Satisfaction, mean (SD)	% intending to continue
	Men	306	8.6 (1.5)	99.3

Note. Reported descriptively; no hypothesis tests were conducted. There were 49 respondents who did not select "yes" to intended six-month continuation overall (34 women, 15 men), of which 20 occurred in the ≤3-month band (16 women, 4 men). Abbreviations: mo, months; yr, years.

Table S7. Baseline characteristics by gender and duration band.

Duration	Gender	n	Age, mean (SD)	BMI, median (IQR)	Activity, mean (SD)	Dose, median (IQR)	Loading %	% once-daily
<1 mo	Women	26	53.5 (13.5)	25.2 (22.5–33.6)	3.2 (0.8)	5.0 (5.0–5.0)	4	88.5
	Men	26	53.1 (11.8)	29.5 (26.9–34.3)	2.6 (1.0)	5.0 (5.0–8.8)	12	73.1
1–3 mo	Women	117	54.6 (11.8)	26.5 (23.1–31.3)	3.0 (0.8)	5.0 (5.0–5.0)	6	79.5
	Men	66	56.8 (12.3)	28.6 (25.7–32.0)	3.0 (0.8)	5.0 (5.0–10.0)	23	71.2
3–6 mo	Women	174	54.5 (11.9)	25.5 (22.0–30.3)	3.3 (0.7)	5.0 (5.0–5.0)	6	84.5
	Men	109	54.3 (12.2)	27.3 (24.8–32.2)	3.1 (0.7)	5.0 (5.0–10.0)	11	77.1
6–12 mo	Women	319	54.6 (10.1)	25.1 (21.9–29.6)	3.3 (0.7)	5.0 (5.0–5.0)	15	85.6
	Men	231	54.5 (13.4)	27.6 (24.4–32.3)	3.3 (0.7)	5.0 (5.0–10.0)	19	79.2
1–3 yr	Women	344	53.3 (10.4)	24.4 (21.8–29.4)	3.4 (0.7)	5.0 (5.0–8.0)	15	81.7
	Men	301	53.1 (12.1)	26.9 (24.8–31.4)	3.4 (0.6)	6.0 (5.0–10.0)	23	77.7
3+ yr	Women	75	52.3 (12.4)	23.1 (21.0–27.5)	3.7 (0.7)	5.0 (5.0–10.0)	19	89.3
	Men	306	48.9 (12.1)	26.7 (24.7–29.8)	3.6 (0.6)	7.0 (5.0–10.0)	22	69.9

Note. Values are within duration band and gender. Age and activity are mean (SD); BMI and daily dose are median (IQR). Loading % is the share reporting an initial loading protocol. Cycling users are not shown. Group n refers to the retained analytic sample; dose summaries use the dose-valid subset (Table S3), whereas the percentage taking creatine once daily is computed on the frequency-valid sample (dosing frequency was complete for all respondents). Doses > 30 g/day were set to missing for dose-specific summaries only. Abbreviations: mo, months; yr, years.

Table S7, panel B: physical-activity types by gender.

Physical-activity type	Women %	Men %
Strength training / weightlifting	82.7	86.7
General fitness / cardio	64.8	62.7
Endurance sports (running, cycling, rowing)	20.5	28.0
Team sports	4.5	7.5
No physical activity	1.3	1.7

Note: Percent of eligible (current or recent) binary-gender respondents (n = 2,178) endorsing each activity type; respondents could endorse multiple types. Physical-activity level (1–5) by gender and duration is given in the main S7 panel above; among women it increased across duration categories (3.00 at 1–3 months to 3.65 at 3+ years), and strength-training participation likewise increased across duration categories among women (69.2%, 73.5%, 77.6%, 83.4%, 87.2%, and 90.7% from <1 month to 3+ years; n = 1,055 duration-placeable women). Across the same bands (<1 month to 3+ years), the other activity types showed no clear monotonic trend among women: general fitness or cardio (65.4%, 65.8%, 62.6%, 68.3%, 64.0%, 62.7%), endurance sports (19.2%, 17.9%, 12.1%, 22.9%, 23.3%, 21.3%), team sports (3.8%, 1.7%, 2.9%, 4.4%, 7.3%, 1.3%), and no physical activity (0.0%, 4.3%, 2.3%, 0.9%, 0.3%, 0.0%).

Table S8. Individual knowledge/belief-item endorsement among women across duration categories.

Item	<1 mo	1–3 mo	3–6 mo	6–12 mo	1–3 yr	3+ yr	Men %	p	q
Kidney damage	0 (0.0)	4 (3.4)	5 (2.9)	17 (5.3)	4 (1.2)	0 (0.0)	1.3	0.020	0.102
Hair loss	1 (3.8)	2 (1.7)	2 (1.1)	5 (1.6)	2 (0.6)	0 (0.0)	0.3	0.458	0.764
Is a steroid	0 (0.0)	0 (0.0)	1 (0.6)	2 (0.6)	1 (0.3)	0 (0.0)	0.3	0.894	0.894
Only for lifters	0 (0.0)	2 (1.7)	3 (1.7)	3 (0.9)	6 (1.7)	0 (0.0)	0.7	0.775	0.894
Unsafe long-term	1 (3.8)	0 (0.0)	1 (0.6)	2 (0.6)	3 (0.9)	0 (0.0)	0.5	0.354	0.764

Note: Cell entries are the number and percentage of women endorsing the incorrect ("true") response, n (%); n per band (women) = 26, 117, 174, 319, 344, and 75. Men's overall endorsement is shown for context. The across-band test is a within-women chi-square across the six ordered duration bands (Monte Carlo p where any expected cell < 5), Benjamini–Hochberg corrected within this belief-item family. No item differed across duration after correction (all q ≥ 0.102). Abbreviations: mo, months; yr, years.

Table S9. Covariate-adjusted ordered duration trends in goals and information-source endorsement among women (n = 1,016).

Item	Covariate-adjusted ordered trend			Unadjusted categorical association
	Adjusted OR per 1-category increase	95% CI	q (BH)	Cramér's V
**Goals (% endorsing)**				
Healthy aging	1.08	0.97–1.21	0.248	0.095
Cognition	1.06	0.95–1.19	0.315	0.072
Energy	0.89	0.80–1.00	0.149	0.063
General wellness	1.04	0.94–1.16	0.440	0.085
Athletic performance	1.07	0.95–1.20	0.314	0.104
Strength	1.11	0.99–1.23	0.149	0.121
Muscle mass	1.15	1.03–1.28	0.093	0.126
Exercise recovery	1.11	0.99–1.25	0.149	0.126
Weight management	0.88	0.75–1.03	0.206	0.080
**Information sources (% reporting)**				
Coach or trainer	1.38	1.18–1.62	<0.001	0.154
Friend or family	0.83	0.73–0.95	0.018	0.120
Social media	0.94	0.83–1.05	0.491	0.102
YouTube	1.02	0.82–1.28	0.829	0.060
Healthcare provider	0.95	0.81–1.13	0.660	0.070
Podcasts	1.10	0.99–1.24	0.205	0.104
Research articles	1.04	0.92–1.17	0.660	0.060

Note. Analyses are restricted to women with complete data for age, BMI, physical-activity level, and the modeled outcome (n = 1,016). Reported duration of creatine use was coded as a six-level ordered variable: 1 = <1 month, 2 = 1–3 months, 3 = 3–6 months, 4 = 6–12 months, 5 = 1–3 years, and 6 = 3+ years. Adjusted odds ratios represent the change in odds of endorsing the listed goal or information source associated with a one-category increase toward longer reported duration of use, controlling for age, BMI, and physical-activity level; an odds ratio above 1 indicates greater endorsement with longer reported duration and below 1 indicates lower endorsement. The reported q-values are Benjamini–Hochberg-adjusted p-values from these covariate-adjusted ordered-trend models, corrected separately within the goal and information-source domains.

Cramér's V is the effect size from the corresponding unadjusted six-category omnibus comparison and is not derived from the adjusted regression model.

Table S10. Sensitivity analyses for the confidence and knowledge-alignment measures.

Specification	Estimate	95% CI	p
**Panel A. Confidence-model robustness (gender × knowledge interaction)**			
Primary linear model (β)	−0.096	−0.171, −0.022	0.012
HC3 robust SE (β)	−0.096	−0.170, −0.022	0.013
Proportional-odds ordinal (interaction cumulative OR)	0.83	0.70, 0.97	0.022
PO assumption test (interaction term)	—	—	0.33
Categorical knowledge specification vs linear (LRT)	—	—	0.75
**Panel B. Bounded knowledge-alignment score models (gender, women vs men)**			
Linear (β)	−0.293	−0.388, −0.198	<0.001
Proportional-odds ordinal (cumulative OR)	0.488	0.397, 0.598	<0.001
Quasi-binomial on k/5 (OR)	0.540	0.448, 0.652	<0.001
**Panel C. Item-response-theory scoring sensitivity (standardized gender difference)**			
Unweighted sum	−0.34	—	<0.001
Rasch latent trait	−0.35	—	<0.001
Two-parameter logistic latent trait	−0.35	—	<0.001
Correlation of sum with latent trait	0.984 (Rasch); 0.971 (2PL)	—	—

Note: Panel A: the primary self-rated-confidence model is linear with the gender × knowledge-alignment interaction; robustness is assessed with HC3 heteroscedasticity-robust standard errors and a proportional-odds ordinal refit (n = 2,055). Term-specific score tests did not indicate proportional-odds violations for the interaction term (p = 0.33) or the knowledge slope (p = 0.26), although the omnibus test indicated a global departure from proportional odds. Residual diagnostics for the primary linear model: residuals were approximately symmetric and near-normal (skewness 0.17, excess kurtosis 0.00) with no evidence of functional-form misspecification (Ramsey RESET p = 0.10) but detectable heteroscedasticity (Breusch–Pagan p = 0.002), which is why HC3-robust standard errors are reported and the interaction is additionally checked with the proportional-odds ordinal model. Panel B: the bounded 0–5 knowledge-alignment score modelled three ways, each adjusted for duration, age, physical-activity level, and BMI (n = 1,934). Panel C: the five items scored as an unweighted sum versus Rasch and two-parameter logistic latent traits; standardized gender coefficients are from age-adjusted models (n = 2,139).

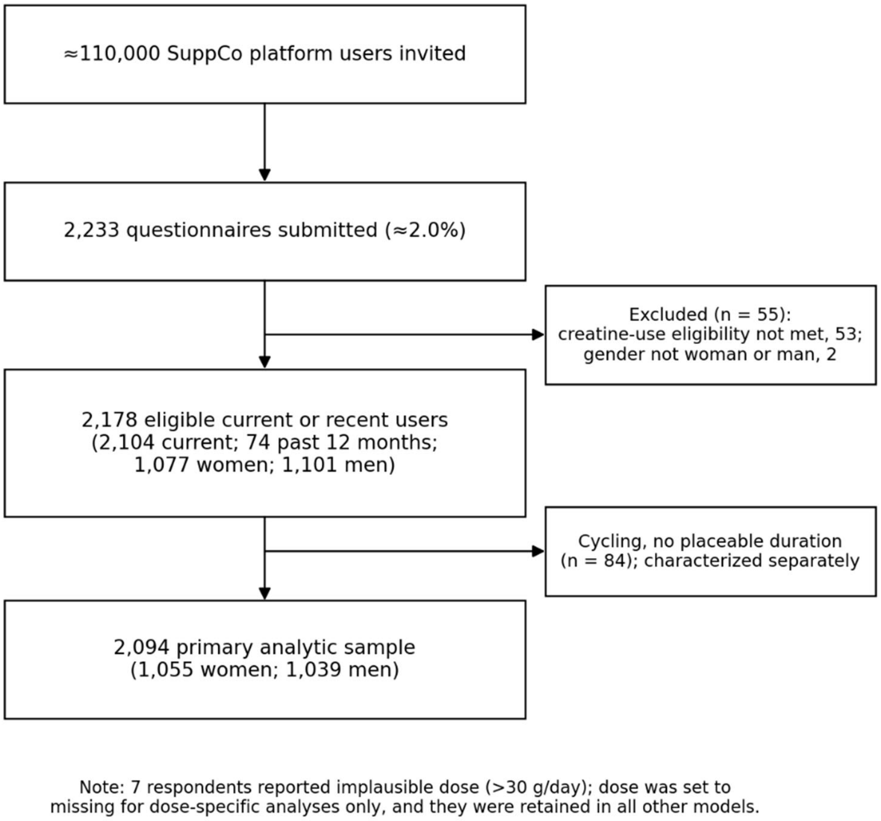

Figure S1. Participant flow of included analytic sample.
